# Efavirenz-Altered Gut-Microbiota, *Tph1*, and Systemic 5-HT Levels: Could They Affect Depression Mechanisms in Mice?

**DOI:** 10.3390/ijms27104504

**Published:** 2026-05-18

**Authors:** Sandra Angélica Rojas-Osornio, Vladimir Paredes-Cervantes, María Magdalena Aguirre-García, Minerva Crespo-Ramírez, Claudia C. Márquez-Mota, Raquel Aguilar-Rosales, José Moisés Talamantes-Gómez, Alma Reyna Escalona-Montaño, Águeda García-Pérez, Miguel Pérez de la Mora, Dasiel O. Borroto-Escuela, Leticia Manuel-Apolinar, Gilberto Pérez-Sánchez, Salvador Vazquez-Vega, Ricardo Martínez-Lara, Emiliano Tesoro-Cruz

**Affiliations:** 1 Academia de Bioquímica Médica II, Departamento de Formación Básica Disciplinaria, Escuela Superior de Medicina, Instituto Politécnico Nacional, Mexico City 11340, Mexico; sandii38@yahoo.com.mx; 2 Laboratorio Central, Hospital de Especialidades “Dr. Antonio Fraga Mouret”, Centro Médico Nacional “LaRaza”, Instituto Mexicano del Seguro Social, Mexico City 02990, Mexico; vlapace@hotmail.com; 3 Unidad de Investigación UNAM-INC, División de Investigación, Facultad de Medicina, Instituto Nacional de Cardiología Ignacio Chávez, Universidad Nacional Autónoma de México, Mexico City 14080, Mexico; maguirre@unam.mx (M.M.A.-G.); rar22700@gmail.com (R.A.-R.); almaescalona@comunidad.unam.mx (A.R.E.-M.); 4 Division de Neurociencias, Instituto de Fisiología Celular, Universidad Nacional Autónoma de México, Mexico City 04510, Mexico; mcrespo@ifc.unam.mx (M.C.-R.); mperez@ifc.unam.mx (M.P.d.l.M.); 5 Departamento de Nutrición Animal y Bioquímica, Facultad de Medicina Veterinaria y Zootécnia, Universidad Nacional Autónoma de México, Mexico City 02990, Mexico; c.marquez@unam.mx (C.C.M.-M.); josetalamantesg@fmvz.unam.mx (J.M.T.-G.); aguedagp@unam.mx (Á.G.-P.); 6 Receptomics and Signaling Networks in Brain Diseases (Group C22), Instituto de Investigación Biomédica de Málaga y Plataforma en Nanomedicina−IBIMA Plataforma BIONAND., 29010 Malaga, Spain; dasiel@uma.es; 7 Receptomics & Brain Disorders Laboratory, Department of Human Physiology and Physical Education and Sport Sciences, School of Medicine, University of Malaga, 29010 Malaga, Spain; 8 Unidad de Investigación Médica en Enfermedades Endocrinas, Hospital de Especialidades, Centro Médico Nacional “Siglo XXI”, Instituto Mexicano del Seguro Social, Mexico City 06720, Mexico; leticia.manuel@imss.gob.mx; 9 Laboratorio de Psicoinmunología, Dirección de Investigaciones Biomédicas en Salud Mental, Instituto Nacional de Psiquiatría Ramón de la Fuente Muñiz, Mexico City 14370, Mexico; gilberto.perez.sanchez.1981@gmail.com; 10 Unidad de Investigación Epidemiológica y en Servicios de Salud, Centro Médico Nacional “Siglo XXI”, Instituto Mexicano del Seguro Social, Mexico City 06720, Mexico; salvazvega@gmail.com; 11 Unidad de Investigación Biomédica en Inmunología e Infectología, del Hospital de Infectología del Centro Médico Nacional “La Raza” IMSS, Mexico City 02990, Mexico; rml900_z@hotmail.com

**Keywords:** efavirenz, gut microbiota, tryptophan hydroxylase type 1, serotonin, short chain fatty acids, depression

## Abstract

The gut microbiota produces molecules that trigger responses at the local and distant levels. It affects the brain through several metabolic products, including serotonin (5-HT). Tryptophan hydroxylase type 1 (Tph1) is the rate-limiting enzyme during 5-HT biosynthesis in the gut. Efavirenz (EFV), an antiretroviral agent against HIV, is associated with depression disorders and Tryptophan hydroxylase type 2 (*Tph2*) deregulation in mice. The possible association between the depressive effects of EFV secondary to dysbiosis and the expression of *Tph1* in the intestine is yet to be studied. Therefore, we aimed to elucidate the role of the gut microbiota in depression mechanisms. We reviewed the gut microbiota, their metabolites (short-chain fatty acids [SCFA]), *Tph1* expression in the gut, and systemic 5-HT and tryptophan levels in CD1 mice after 36 days of oral EFV (10 mg/kg) treatment. The proportions of *Bacteroidota* and *Bacillota_A_368345* decreased and increased, respectively, following EFV treatment. Additionally, the abundance of *Lactobacillus* spp. and *Faecalbaculum* decreased, whereas that of *Dubosiella* spp., *Blautia_A_141780*, and *Anaerostipes* increased. These bacteria contribute to SCFA production and may have counteracted the lack of protective effects provided by *Lactobacillus*. *Tph1* expression was dysregulated in the gut, whereas serum 5-HT levels decreased following EFV treatment. *Lactobacillus* species promote 5-HT production in the gut, and the deregulation of *Tph1* affects 5-HT synthesis. This disruption in the gut–brain axis decreased peripheral 5-HT levels. This affects the serotonergic system in the brain, which could contribute to depression.

## 1. Introduction

The gut microbiota is considered a virtual endocrine organ. It produces molecules that can interact with the host physiology to trigger responses at local and distant levels, owing to its bidirectional role in regulating the function of several organs. Multiple factors affect its composition (diet, lifestyle, and drugs). Alterations in the composition of the intestinal microbiota (dysbiosis) are associated with extra-digestive diseases, such as metabolic, immunological, cardiovascular, and neuropsychiatric disorders [1,2,3,4,5,6,7,8,9,10].

The presence of certain bacteria is associated with inflammatory molecules that can trigger a cascade of inflammatory pathways involving immune cells, such as macrophages and neutrophils, producing pro-inflammatory factors, including interleukins and other cytokines, which are triggered by bacterial structural elements (such as lipopolysaccharide) [11]. Similarly, byproducts of bacterial metabolic processes, including short-chain fatty acids (SCFAs) such as butyrate, propionate, and acetate, can help inhibit inflammatory processes [11,12].

Dysbiosis in the brain facilitates the release of neurotransmitters and cytokines that cause neuroinflammation and are related to the pathogenesis of neuropsychiatric diseases [13] and depressive symptoms. These inflammatory molecules are associated with a decrease in distinct microbial groups, such as the genera *Dialister*, *Bifidobacterium*, *Coprococcus*, and *Lactobacillus* [14,15].

The biological mechanisms underlying the depressive effects of efavirenz (EFV) secondary to dysbiosis remain largely understudied. However, many of these bacteria are involved in the synthesis of glutamate, butyrate, serotonin (5-HT), and gamma-aminobutyric acid (GABA), which are key to the mechanism of depression [16,17].

Gao et al. [18,19] revealed that changes in the gut microbiota composition affect the gut–brain axis by modulating tryptophan metabolism. Products of tryptophan metabolism, such as 5-HT, have profound effects on the interaction between the gut microbiota and the gut–brain axis [20,21].

EFV, a non-nucleoside reverse transcriptase inhibitor, has been the first-line component of antiretroviral therapy (ART) worldwide for several years. However, 25–40% of treated people living with HIV (PLWH) have reported abnormal dreams, insomnia, mood disturbances, nervousness, anxiety, depression, and dizziness in clinical studies; similar results have been observed in murine models [22,23,24]. Moreover, this drug affects brain energy metabolism dysfunction, especially in the cerebral cortex, striatum, and hippocampus of mice [25,26]. Alterations in monoamine turnover, as well as GABA and glutamate levels, have also been reported in the rat striatum after oral administration of EFV [27].

Acute administration of EFV damages the blood–brain barrier (BBB) through various secondary mechanisms; it induces endoplasmic reticulum stress, along with mitochondrial and autophagy dysfunction [28]. Huang et al. [28] also observed a significant increase in the levels of proinflammatory cytokines (IL-6, IL-1β, and IL-8) and NOS2 in the brain after 24 and 48 h of exposure to EFV. Similarly, we previously reported Tryptophan hydroxylase type 2 (Tph2) dysregulation in the brainstem, amygdala, and hypothalamus of mice after chronic oral administration of EFV, which resulted in increased 5-HT levels in the amygdala [29].

Ray et al. [30] reported the effects of EFV on the intestinal microbiota. High concentrations inhibited the growth of *Enterococcus faecalis* (100%), Bacteroides (75%), and Prevotella (100%) [30]. Furthermore, ART did not reverse HIV-induced gut microbiome dysbiosis and may have aggravated microbiota alterations, owing due to the antibacterial effect of EFV [30]. Research suggests that EFV damages intestinal crypts, reduces the number of protective goblet cells, and lowers the expression of intestinal tight junction molecules, such as zonula occludens-1. EFV causes gut dysbiosis and significantly increases gut permeability [31].

Tryptophan hydroxylase 1 (Tph1) is a crucial enzyme for 5-HT biosynthesis in the peripheral tissues of the body, such as the intestine and skin. It differs from tryptophan hydroxylase 2 (Tph2), which is mainly found in the brain. This enzyme catalyzes the rate-limiting step in 5-HT synthesis by converting the amino acid tryptophan (Trp) to 5-hydroxytryptophan [32,33,34].

The possible association between the depressive effects conferred by EFV secondary to dysbiosis and the expression of Tph1 in the intestine remains largely understudied. Therefore, the aim of this study was to investigate alterations in the gut microbiota, their metabolites (short-chain fatty acids, SCFAs), Tph1 expression in the gut, and systemic 5-HT and Trp levels after oral administration of EFV to assess its role in the mechanism of depression in mice.

## 2. Results

### 2.1. Characterization of the Gut Microbiota

**Figure 1:**
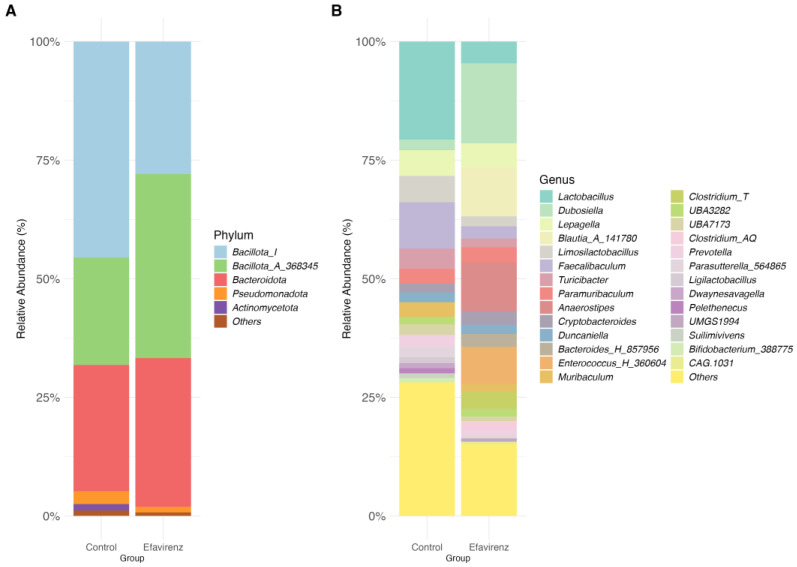
Taxonomic composition of the gut microbiota of CD1 mice. Taxonomic composition of the gut microbiota in an experimental murine model comparing two groups (control and efavirenz (EFV), *n* = 4 per group). Relative abundance (%) at the phylum (**A**) and genus (**B**) levels is presented. Only taxa with relative abundances >1% are shown.

Taxonomic composition of the microbiota at the phylum and genus level following oral administration of EFV in CD1 mice is shown in Figure 1. At the phylum level, the EFV group had a lower proportion of *Bacteroidota* and a higher proportion of *Bacillota_A_368345* than the control group. At the genus level, a decrease in *Lactobacillus* spp. and *Faecalbaculum*, and an increase in *Dubosiella* spp., were observed in the EFV group compared to those in the control group. *Blautia_A_141780* and *Anaerostipes* were also detected in the EFV group (Figure 1).

### 2.2. Comparative Analysis of Intestinal Microbial Diversity

**Figure 2:**
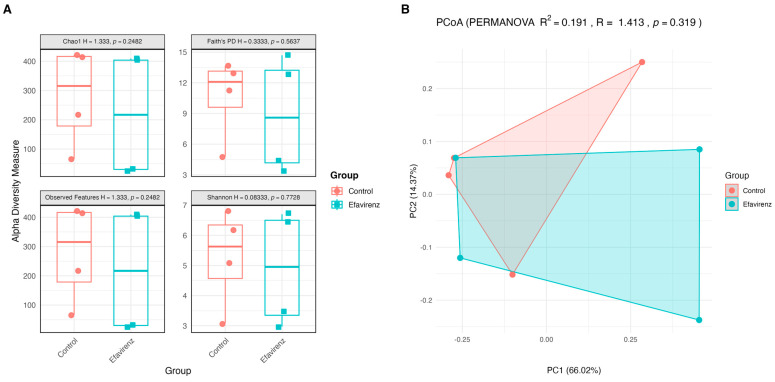
Comparative analysis of intestinal microbial diversity in the control and EFV groups. (**A**) Alpha diversity assessed using the Shannon, Faith’s Phylogenetic Diversity (Faith PD), Chao1, and Observed Features indices. (**B**) Beta diversity estimated with the Bray–Curtis index and represented by a principal coordinate analysis (PCoA), accompanied by the permutational multivariate analysis of variance result (*n* = 4 per group).

Comparative analysis of intestinal microbial diversity between the control and EFV groups showed no significant differences in alpha and beta diversity (Figure 2).

### 2.3. Exploratory Analysis Using Heat Maps

**Figure 3:**
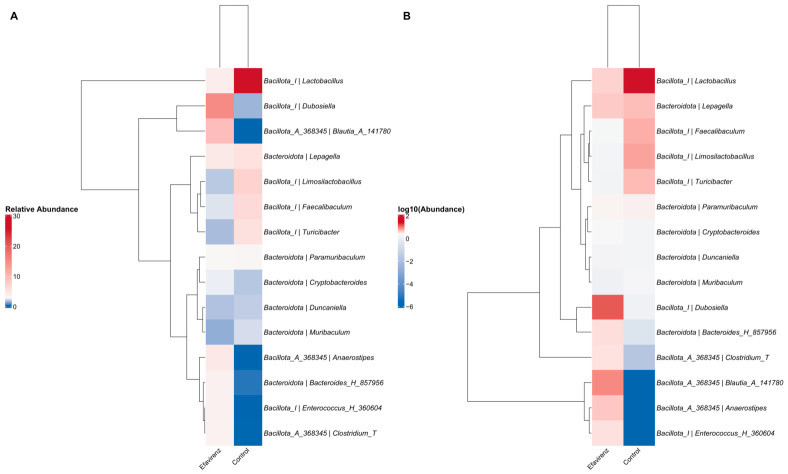
Correlation analysis of bacterial genera and experimental variables using heat maps. (**A**) A heatmap showing the correlation using the relative abundance of bacterial genera to identify associations with the experimental groups. (**B**) A heatmap based on abundance transformed with a base-10 logarithm, used to minimize the effect of extreme values and facilitate the detection of patterns in the correlations (*n* = 4 per group).

Correlation analysis of the bacterial genera and experimental variables using heat maps revealed differences in the abundance profiles of some bacterial genera. Dendrograms show the hierarchical grouping of taxa according to abundance profiles. In particular, the most abundant phylum was Bacillota. At the genus level, *Lactobacillus* and *Dubosiella* were more abundant in the control and EFV groups, respectively. However, these variations between groups were not statistically significant (Figure 3).

### 2.4. Short Chain Fatty Acids (SCFA)

**Figure 4:**
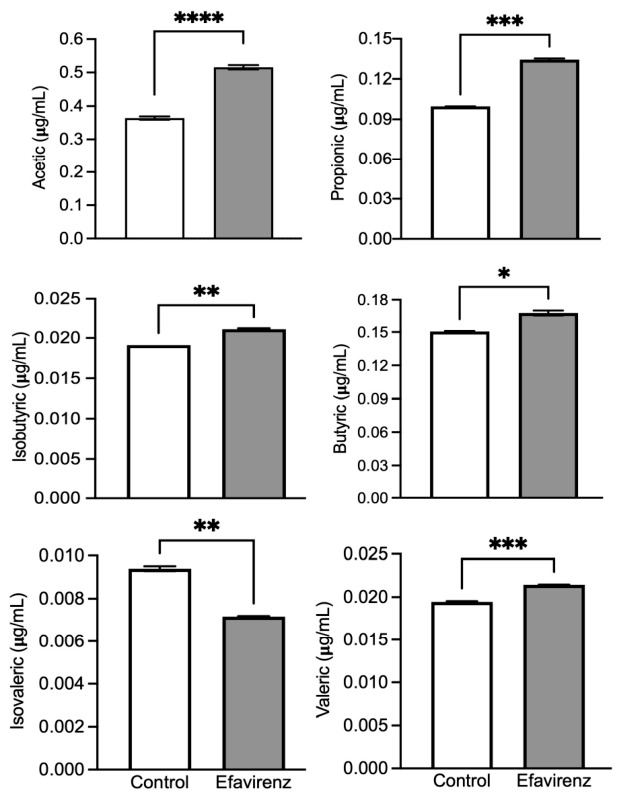
Short-chain fatty acids detected in the feces of mice treated with EFV. Acetic (F_2,2_ = 1.747; **** *p* < 0.0001), propionic (F_2,2_ = 77.47; *** *p* < 0.001), isobutyric (F_2,2_ = 1922; ** *p* < 0.01), butyric (F_2,2_ = 20.96; * *p* < 0.05), and valeric (F_2,2_ = 28.44; *** *p* < 0.001) acid contents increased in mice treated with EFV, whereas isovaleric acids levels decreased (F_2,2_ = 15.60; ** *p* < 0.01) (Welch’s *t* test; *n* = 3).

Figure 4 shows the SCFA contents of mice treated with EFV and control groups (*n* = 3 per group). All SCFA concentrations increased in the EFV group, except for that of isovaleric acid, which decreased (Welch’s *t*-test, F = 15.60_2,2_; *p* < 0.01).

### 2.5. Effect of EFV on Tph1 Expression in the Gut, and on Serum 5-HT and Trp Levels

**Figure 5:**
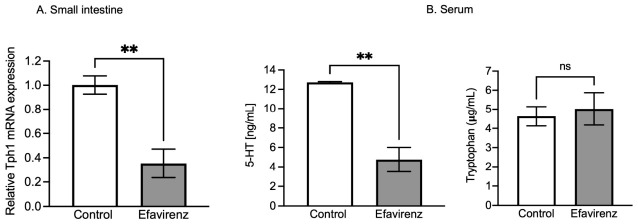
Effect of EFV on tryptophan hydroxylase type 1 (*Tph1*) expression in the gut (**A**); serum 5-HT and Trp levels (**B**) in CD1 mice. *Tph1* mRNA expression was diminished following EFV administration compared to that in the control group (**A**) (F_2,2_ = 2.443; ** *p* < 0.01). 5-HT levels were diminished after 36 days of EFV treatment (**B**). Unpaired t test with Welch’s correction ** *p* < 0.01. To elucidate the likely functional correlation between *Tph1* expression and 5-HT synthesis, the serum levels of this neurotransmitter and the quantity of Trp were measured in mice. An unpaired *t* test with Welch’s correction revealed a statistically significant decrease in the 5-HT level after EFV treatment (F_2,2_ = 229, *p* < 0.01), although serum Trp levels did not change (F_4,4_ = 2.822, *p* > 0.05) (**B**); ns = non-significance.

Real-time polymerase chain reaction (qPCR) was performed after 36 days of antiretroviral treatment to determine *Tph1* mRNA expression (*n* = 3), whereas ELISA was performed to determine serum 5-HT (*n* = 3) and Trp (*n* = 5) levels in CD1 mice. Unpaired t-tests with Welch’s correction analysis revealed significant differences in *Tph1* expression after EFV (F_2,2_ = 2.443; *p* < 0.01) (Figure 5A) and 5-HT (F_2,2_ = 229, *p* < 0.01) treatments (Figure 5B). Trp levels did not differ between groups (F_4,4_ = 2.822; *p* > 0.05) (Figure 5B).

## 3. Discussion

EFV is used to treat HIV. However, it causes depression and other neuropsychiatric effects such as anxiety, insomnia, vivid dreams, and depression [24,27]. Although its main function is to block HIV replication, this drug has a considerable effect on the central nervous system (CNS), demonstrating its ability to cross the BBB [28].

However, the mechanism by which EFV causes depression remains unclear. Several studies have sought to elucidate these mechanisms. For instance, Zareifopoulos et al. [35] reported interactions with 5-HT receptors such as 5-HT2A. The chemical structure of EFV is similar to that of hallucinogenic compounds such as lysergic acid diethylamide, which are closely linked to the regulation of mood and perception [35].

Apostolova et al. [36] reported alterations in the function of mitochondria in neurons, causing toxicity. Moreover, cellular energy production and oxidative stress processes are affected in the brain. Cavalcante et al. [37] reported destabilization of the neurotransmitter systems of 5-HT or norepinephrine, unlike common antidepressants that increase the levels these neurotransmitters, leading to anxiety, insomnia, and depression. Furthermore, other studies have suggested that certain byproducts of EFV (active metabolites) that form in the body are more toxic to brain cells than the drug itself [38]. Similarly, our group reported that EFV triggers the dysregulation of *Tph2* in the three serotonergic areas studied, and that 5-HT levels increased in the amygdala. Anxiety and depression-like behaviors have also been observed in mice [29].

Animal and human studies of the microbiome after EFV administration, particularly in the context of ART, have indicated that EFV-based regimens induce considerable gut dysbiosis. This dysbiosis is characterized by reduced bacterial diversity, specific microbial shifts, and compromised integrity of the intestinal barrier [30]. When gut dysbiosis occurs, it generally triggers a pro-inflammatory state characterized by increased intestinal permeability; elevated systemic inflammatory markers, including C-reactive protein, lipopolysaccharides, and LPS-binding protein; and heightened inflammatory cytokines as IL-6, TNF-alpha, IL-1β, and IL-17. Moreover, beneficial bacteria that produce anti-inflammatory compounds decrease whereas harmful bacteria (pathobionts) flourish and release toxins that trigger systemic inflammation [39].

These changes are often more pronounced with newer agents such as dolutegravir (DTG). In a recent study by Huang et al. [31] EFV treatment in mice resulted in the destruction of intestinal crypts, loss of goblet cells, and more severe damage to gut barrier integrity than in the DTG and normal control groups. In contrast, the same research group identified a significant reduction in *Lactobacillus* abundance in EFV-treated mice. This was consistent with our results in which mice from the EFV group showed a decrease in *Lactobacillus* spp. (Figure 1B). Moreover, murine studies have revealed a correlation between a reduction in Lactobacillus species and increased neuroinflammation, higher levels of kynurenine, and behavioral despair. Conversely, dietary supplementation with *Lactobacillus* can reverse these behaviors and reduce neuroinflammation [40,41].

Several studies have identified the mechanisms by which *Lactobacillus* affects depression and directly acts on the brain: (i) neurotransmitter regulation—some *Lactobacillus* strains facilitate the synthesis of essential chemicals such as 5-HT and dopamine [42]; (ii) stress management—*Lactobacillus* help regulate the hypothalamic–pituitary–adrenal axis, which reduces cortisol levels, consequently improving resilience to stressful situations [43]; (iii) inflammation reduction—which is often linked to systemic inflammation. *Lactobacillus* helps maintain the integrity of the intestinal barrier, thereby preventing inflammatory substances from entering the bloodstream and affecting the brain [44]; and (iv) GABA receptor modulation—specific *Lactobacillus* strains minimize neuronal activity and reduce anxiety [45].

The gut microbiota, particularly *Lactobacillus*, enhances 5-HT synthesis by modulating Trp metabolism. Trp is a biosynthetic precursor of several microbial metabolites [46]. A decrease in Lactobacillus in the EFV group (Figure 1B) impaired this process, leading to lower systemic 5-HT levels (Figure 5B). This is consistent with the findings of Da Fonseca et al. [47], who reported a connection of the Trp–5-HT–Microbiota Axis. These results indicate that the gut microbiota, particularly *Lactobacillus* and *Bifidobacterium*, enhances 5-HT synthesis by modulating Trp metabolism. In the present study, we evaluated systemic Trp levels in mice but did not observe changes between groups (F_4,4_ = 2.822, *p* > 0.05) (Figure 5B). However, we expected an increase in Trp levels because *Tph1* was dysregulated, possibly resulting in an excess of Trp (because it had not been used for 5-HT synthesis). Therefore, Trp may have been insufficiently detected during ELISA, although it showed a slight upward trend (Figure 5B). This could be considered a limitation of our study and suggests a need for the measurement of Trp using HPLC in future studies.

In addition to a decrease in the abundance of *Lactobacillus* spp., we detected a decrease in *Faecalbaculum* in the EFV group. *Faecalbaculum* is a genus of Gram-positive, strictly anaerobic, non-motile bacteria belonging to the family Erysipelotrichaceae. Its principal species is *Faecalibaculum rodentium*, which prevents intestinal tumors by regulating epithelial homeostasis in mice. Zagato et al. [48] reported that *F. rodentium* is an endogenous member of the murine microbiota. It can prevent the growth of intestinal tumors in both mice and humans, highlighting its potential for translational applications [48].

Lower and higher proportions of *Bacteroidota* and *Bacillota_A_368345*, respectively, were observed in the EFV group. The low abundance of *Bacteroidota* in the gut microbiota is often referred to as dysbiosis and is commonly associated with obesity, inflammatory bowel disease, and reduced dietary fiber breakdown. These bacteria are essential for gut health, and their decline can reduce the ability to metabolize fats and complex sugars, thereby affecting metabolic health. This is consistent with our recent study, wherein EFV-treated mice showed a decrease in body weight. This occurred despite increased food intake resulting from appetite stimulation by specific compounds, hormones, and neural signals acting on the brain’s hunger centers, primarily in the hypothalamus, to promote eating behaviors. Furthermore, an increase in serum triglyceride and cholesterol levels was detected [49].

*Bacillota* spp. are involved in nutrient fermentation, production of SCFAs such as acetate and butyrate, and regulation of intestinal metabolism. Their participation also affects gut–brain communication and metabolic, immunological, and neurological processes [50]. This concept could be related to our results, in which all SCFA (acetic, propionic, isobutyric, butyric, and valeric acids) measured increased in the EFV group, with the exception of isovaleric acid (Figure 4).

SCFAs are the products of bacterial fermentation in the colon and are directly related to bacterial growth. They have different effects on genes that regulate cell proliferation, the cell cycle, and anti-inflammatory effects [51], which constitute a link in distant organs. The observed increase in acetic acid (Figure 4), a product of the alcoholic fermentation of bacteria (for example, *Bacteroides*), could be related to oxidative stress—a known effect of EFV on neurons. Acetate can generate oxidative stress and increase the sympathetic response [52]. The increase in propionic acid levels (Figure 4) could be related to the EFV-induced emotional imbalance at the amygdala level. In a study on the effect of acute administration of low doses of propionic acid on social behavior, anxiety-like behavior, and the structure/ultrastructure of the central nucleus of the amygdala in adolescent male Wistar rats, even a single and relatively low dose of propionic acid was sufficient to produce fast and relatively long-lasting (48 h after treatment) decreases in social motivation and structural alterations in the amygdala [53].

The final numbers in ‘*Bacillota_A_368345*’ (i.e., 368345) often act as a unique identifier of a group of uncultured or poorly characterized bacteria within the class Clostridia or Bacilli in sequencing databases (such as EzBioCloud or NCBI) for a specific strain, operon, or taxon in 16S rRNA analysis. Aires and Souza [54] examined the composition of the microbial community. Specific taxa under the designation “*Bacillota A 368345*” were identified, suggesting an adaptation to specific organic matter conditions in industrial wastewater. However, no precise information was found to explain the increase in this bacterium. Therefore, further research is required.

Our results also indicated an increase in *Dubosiella* spp., *Blautia_A_141780*, and *Anaerostipes* compared with those in the control group (Figure 1A). *Dubosiella* spp. participate in nutrient metabolism, thereby contributing to the production of bacterial metabolites, such as butyrate and acetate, which improve the symptoms of depression and anxiety. It is considered a beneficial bacterium within the gut–brain axis [55]. This aligns with our findings regarding the increases in acetate and butyrate levels (Figure 4).

*Blautia* can ferment fiber and produce acetate, butyrate, and lactate. These metabolites have anti-inflammatory properties that protect the blood–brain barrier. Additionally, this bacterium affects the production of neurotransmitters that are essential for mood and helps reduce systemic inflammation—a factor closely linked to the pathophysiology of depression [56]. Specifically, higher levels of *Blautia_A_141780* were often associated with better mental health and fewer depressive symptoms. People diagnosed with major depressive disorder often show a significant decrease in *Blautia* abundance compared to healthy individuals [56,57].

Anaerostipes, a butyrate-producing bacterium, is a beneficial component of the gut microbiota. It belongs to the Lachnospiraceae family (*Firmicutes phylum*) and is highly regarded for its metabolic flexibility and ability to ferment carbohydrates, lactate, and acetate to produce butyrate in the colon. Butyrate exhibits potent anti-inflammatory properties and protects the intestinal barrier. Thus, its decline has been directly linked to the presence of depressive symptoms [58]. Moreover, a reduction in the levels of butyrate-producing bacteria is frequently observed in patients with depression [58,59]. This is consistent with our findings that showed an increase in SCFA concentrations (Figure 4).

In summary, the low proportion of *Lactobacillus* in this study may have been offset by an increase in other bacterial groups, such *Dubosiella*, *Blautia*, and *Anaerostipes*. These bacteria contribute to the production of metabolites such as propionate, butyrate, and acetate, which improve the symptoms of depression and anxiety. These bacterial groups likely counteracted the lack of protective effects of *Lactobacillus* (neurotransmitter regulation, stress management, inflammation reduction, maintaining the integrity of the intestinal barrier, and preventing brain damage). Although the underlying biological mechanisms remain largely understudied, many of these bacteria are involved in the synthesis of glutamate, butyrate, 5-HT, dopamine (DA), and GABA, which are key neurotransmitters in depression [16]. These neurotransmitters are essential transducers of the gut–brain axis and play critical roles both peripherally and centrally.

DA plays a complex, indirect role in enhancing EFV’s impact on the BBB and HIV entry into the CNS, primarily by increasing BBB permeability and promoting the migration of infected immune cells. Although DA does not readily cross the BBB, elevated extracellular DA levels increase BBB permeability and HIV-infected cell transmigration [60].

Regarding our SCFA results coincided with increases in the abundance of bacterial groups, such *Dubosiella*, *Blautia*, and *Anaerostipes*, which contribute to the production of metabolites such as propionate, butyrate, and acetate. However, the correlation analysis of bacterial genera and experimental variables using heat maps (Figure 3) showed no statistically significant differences.

In the present study, Tph1 deregulation reduced peripheral 5-HT levels in EFV-treated mice (Figure 5B). This indicates the failure of the serotonergic system, likely driven by gut-level *Tph1* deregulation and microbial shifts. The signal may reach the CNS via SCFAs and not the produced neurotransmitters. However, these can act on neurons in the digestive tract, consequently regulating mood, anxiety, and depression. Peripheral 5-HT also increases nutrient absorption and storage, regulates the composition of the gut microbiota, and participates in mediating neuronal disorders [61].

In addition, we previously reported the deregulation of Tph2 in the brains of mice after EFV administration [29], including behavioral assays, such as the forced swim test, in which mice revealed a significant increase in immobility time in the EFV-treated group [29], and in the sucrose preference test, mice showed an increased preference for sucrose compared with control mice (day 1: Welch’s *t* test F_4,4_ = 1.114; *p* < 0.01; day 2: Welch’s *t* test F_4,4_ = 1.646; *p* < 0.01) [49]. This information can directly contribute to our understanding of the mechanisms underlying depression in the CNS.

Finally, the gut microbiota affects the brain and may be involved in neuropsychiatric disorders, partly by modulating the availability circulating SCFAs and 5-HT production Tph1 in the gut and Tph2 in the brain. This research was considered a descriptive study, since behavioral experiments were not carried out at the same time as the analytes of this work were measured; however, behavioral experiments with this drug were previously analyzed under the same conditions reported by our group [29,49].

Limitations: The main limitation of this study was the small sample size, which was insufficient to detect significant differences in microbiota analysis. Additionally, we acknowledge the reduced statistical power as a limitation due to the small number of mice per test. Tryptophan (Trp) measurements were performed by ELISA, but this technique was insufficiently sensitive to detect Trp levels compared with HPLC. Therefore, future studies should use more animals to clarify those points and to measure Trp by HPLC.

## 4. Materials and Methods

### 4.1. Mice and Ethical Considerations

Thirty-six mice from a local colony at the Instituto de Fisiología Celular, Universidad Nacional Autónoma de México (UNAM) were used. Adult CD1 male mice (weighing 40–44 g, 12–14 weeks old) were maintained under controlled conditions (12/12 h dark-light cycle, lights on 8:00–20:00, and at a temperature of 22 °C). The mice were divided into two groups: the control and EFV-treated groups, with 18 mice per group (*n* = 36). Mice were housed in groups of six in filter-top cages (17.8 × 30.5 × 12.7 cm) and provided food (LabDiet 5001 PMI., Laboratory Rodent Diet, LabDiet, Hayward, CA, USA) and water ad libitum. This study was conducted in accordance with the Guide for the Care and Use of Laboratory Animals established by Mexican Animal Welfare and Ethical Authorities [62], the Guide for the Care and Use of Laboratory Animals, 8th Edition [63] and the ARRIVE guidelines [64]. The Ethics Animal Experiments Committee of Instituto de Fisiología Celular, UNAM (MPM206-22).and the National Scientific Research Committee (IMSS) (license number R-2021-785-057).

### 4.2. Pharmacological Treatment

The treatment group received EFV (SUSTIVA. tablets, 600 mg; Bristol-Myers Squibb Pharma, Montreal, QC, Canada) (10 mg/kg for 36 days) [65] and the control group received distilled water (1.5 μL/kg). EFV and distilled water were rapidly and gently administered into the mouth using a metal cannula (mouse oral gavage of 3.0 mm diameter, 1.2 mm curve, and 55 mm length for the 18G syringe, Ketu Store, Perth, Australia) attached to a syringe. The animals were weighed daily, and the drug volumes were adjusted to the animal weights to avoid toxic effects. EFV was administered orally once a day for 36 days (for chronic administration) at a dose of 10 mg/kg, as previously reported [61]; and based on the doses used for human therapy (dose: 600 mg daily), as reported previously [29,36,49,66].

### 4.3. Intestinal Preparation Techniques for qPCR Analysis

The procedure used to obtain mouse intestines was performed rapidly to prevent tissue degradation. Each mouse was euthanized using an overdose of ketamine (60 mg/kg) (KETANIL, 10 mL solution; Wildlife Pharmaceuticals, Mexico City, Mexico) and xylazine (6 mg/kg) (PROCIN, 25-mL solution; PiSA Agropecuaria S.A. de C. V., Jalisco, Mexico) via intraperitoneal (IP) injection. The skin of the lower abdomen was lifted with forceps, and a V-shaped or longitudinal incision was made using scissors to expose the peritoneum and abdominal cavity. The stomach and anus were examined to extract the intestines. The junction of the pylori and the ileum terminus was cut, and the small intestine was gently pulled out of the abdominal cavity. Curved forceps were used to grasp and carefully remove the mesentery (the membrane with fat and blood vessels connecting the intestinal loops). A portion of the small intestine was cut below the pyloric sphincter (stomach) and above the cecum to separate the intestine from the colon. After extraction, the intestinal lumen was washed by inserting a needle with a syringe containing cold sterile phosphate-buffered saline at one end of the segment and pressing to remove fecal content and mucus. Each portion of intestine (1.0–1.5 cm) was placed in a 1.5-mL conical microtube with mRNA preservation buffer solution (RNA Stabilization Reagent; Qiagen, Hilden, Germany) (150–250 µL) and stored at −80 °C until use.

### 4.4. RNA Preparation and Tph1 Expression via qPCR Analysis

For qPCR analysis of *Tph1* expression in the gut, frozen gut tissue was powdered and total RNA was extracted using an RNeasy Mini Kit according to the manufacturer’s protocol (Qiagen, Valencia, CA, USA). qPCR analysis was performed using an Applied Biosystems 7300 Real-Time PCR system (Applied Biosystems, Foster City, CA, USA) in a 25 μL reaction mixture containing 100 pg of template RNA. PCR was performed using a One-Step RT- PCR Kit (Applied Biosystems) for Tph1 (Mm01202614_M1) and the beta-actin (Actb) (Mm02619580_G1) TaqMan probes. *Actb* was used as the reference gene. qPCR was performed in triplicate. Reverse transcription was performed at 45 °C for 10 min, followed by inactivation at 95 °C for 10 min and 40 cycles of amplification at 95 °C for 15 s. Annealing was performed at 60 °C for 45 s. The relative amount of mRNA in each sample was calculated using the comparative ΔCt method.

### 4.5. Fecal Sampling for Gut Microbiota Analyses

Fecal sampling from eight mice (four control and four EFV-treated) for microbiota studies (16S rRNA, metagenomics) required strict procedures to avoid cross-contamination and ensure the preservation of microorganisms. This analysis was performed at the end of chronic administration of EFV to mice, including those in the control group. All animals were euthanized with an overdose of ketamine (60 mg/kg) (KETANIL, 10-mL solution; Wildlife Pharmaceuticals) and xylazine (6 mg/kg) (PROCIN, 25-mL solution; PiSA Agropecuaria S.A. de C. V.) via IP injection. Aseptic dissection was performed after the animals were euthanized. Briefly, a fecal sample was obtained directly from the junction of the colon and anus, which was cut. The large intestine was gently obtained, and fecal samples from each mouse were placed in two 1.5 mL cryotubes (800–1000 mg per sample). One microtube was used for each laboratory technique (one for SCFA measurement and another for microbiota analysis). The cryotubes were labeled and immediately placed in a Styrofoam box with dry ice. Subsequently, they were stored at −80 °C until use.

### 4.6. Measurements of Serum Serotonin and Tryptophan Levels by ELISA

Serum serotonin (5-HT) levels were measured using a commercial mouse 5-HT ELISA kit (Cat. No: MBS1601042; My-BioSource, San Diego, CA, USA).

5-HT quantification was performed in triplicate according to the manufacturer’s protocol, and the plate was read spectrophotometrically using an ELISA reader (Thermo Scientific TM, Kennesaw, GA, USA) at 450 nm. The serum 5-HT concentration is expressed in ng/mL.

Serum Trp levels were measured by using a commercial Mouse Trp ELISA kit (Cat. No: MBS008614; My-BioSource, San Diego, CA, USA). Trp quantification was performed in triplicate according to the manufacturer’s instructions, and the plate was read spectrophotometrically at 450 nm using an ELISA reader (Thermo Scientific TM, Kennesaw, GA, USA). The serum Trp concentration is expressed in µg/mL.

### 4.7. DNA Extraction and 16S rRNA Sequencing

DNA was extracted from fecal samples using the QIAamp PowerFecal Pro DNA kit (QIAGEN). The DNA concentration, purity, and integrity were verified using a spectrophotometer (Nanodrop 1000, ThermoFisher, Waltham, MA, USA) and gel electrophoresis (ChemiDoc Image System; Bio-Rad, Hercules, CA, USA). The extracted DNA was sent to Novogene for library preparation and sequencing on a NovaSeq 6000 PE250 platform (Illumina, San Diego, CA, USA). The V3–175 V4 hypervariable region of the 16S rRNA gene was amplified using the primer pairs 341F176 (CCTAYGGGRBGCASCAG) and 806R (GGACTACNNGGTATCTAAT).

### 4.8. Measurement of SCFAs in Fecal Samples

Once the sample was thawed, 500 mg of feces were weighed into a 2.0 mL conical tube and 500 µL of 0.1 M HCl was added, followed by shaking in a minivortex (Heathrow Scientific. SGS, Vernon Hills, IL, USA) for 3 min to integrate the solution with the sample. Next, 500 µL of 0.1 M HCl was added and homogenized with a Scientific Industries SiTM model SI-D238 disruptor, Bohemia, NY 11716 U.S.A. (USA) at 60 Hz for 5 min. The samples were subsequently placed in a Scientific Multifunctional Ultrasonic Cleaner model CS-UB100 ultrasonic bath at a frequency of 40 kHz for 7 min and subsequently centrifuged at 3000 rpm for 15 min in a Heathrow Scientific model Sprout TM centrifuge (Vernon Hills, IL, USA). Finally, the supernatant was filtered using a hydrophilic nylon acrodisc (25 mm diameter, 45 µm pore; Nylon Syringe filter’ Membrane Solutions). The filtered liquid was placed in a 2.0 mL HPLC vial and refrigerated for subsequent injection into the gas chromatograph.

### 4.9. Preparation of the SCFA Curve

A calibration curve was prepared using a WSFA-2 standard (SUPELCO 4-7056; Sigma-Aldrich, Bellefonte, PA, USA). EC Number 23 17912 CAS-No 7732185. for each volatile fatty acid, with a concentration range of 0.2–1.0 µg/µL [67]. The standard contained 0.1% of each volatile fatty acid, equivalent to 1 µg/µL. Six calibration points were prepared (0.1, 0.2, 0.4, 0.6, 0.8, and 1.0 µg/µL). Aliquots of 50, 100, 200, 300, 400, and 500 µL were diluted to 500 µL. Finally, 1 µL from each point was injected.

### 4.10. SCFA Quantification

One microliter (split mode) of the filtrate was injected into an AutoSystem XL gas chromatograph (PerkinElmer Instruments, New York, NY, USA). The operating conditions were as follows: injector temperature, 190 °C; flame ionization detector temperature, 250 °C; oven at 80 °C, with an initial temperature gradient program of 80 °C for 1 min, increasing at 15 °C/min until reaching 200 °C at 4 min; the total run time was 14 min. The carrier gas (nitrogen), hydrogen, and air flow rates were 1, 45, and 450 mL/min, respectively. Volatile fatty acids were quantified using a 30 m long, 0.53 mm diameter DB-FFAP column (PN-125-3232, Agilent J&W GC Columns; Agilent Technologies, Santa Clara, CA, USA) with a film thickness of 1.00 µm.

### 4.11. Bioinformatics and Statistical Analysis

Data analysis was performed using the QIIME2 platform (version 2024.10), complemented by statistical analyses and visualizations in RStudio (version 4.5.1). FASTQ files derived from 16S rRNA gene sequencing were organized and imported into QIIME2. The DADA2 algorithm was applied for noise reduction, error correction, generation of representative sequences, and for filtering out scarce sequences. Taxonomic assignment was performed using a Naive Bayes classifier trained with the Greengenes2 database (version 2024.09) and Scikit-learn (version 1.4.2). Bar charts were generated to represent taxonomic composition at the phylum and genus levels. Representative sequences were aligned using multiple alignments with fast Fourier transform, non-informative regions were masked, and a phylogenetic tree was constructed using FastTree. Shannon, Faith’s phylogenetic diversity, Chao1, and observed feature indices were calculated for alpha diversity analysis. Differences between the groups were assessed using the Wilcoxon signed-rank test. Beta diversity was analyzed using the Bray–Curtis index and permutational multivariate analysis of variance (999 permutations). The distances between communities were visualized using principal coordinate analysis. Rarefaction was performed at a sampling depth of 157,000 sequences/sample. The output results of QIIME2 were complemented with additional analyses in RStudio: relative abundance graphs (most abundant phyla and genera), statistical comparisons between groups (Wilcoxon), heat maps to explore correlations between bacterial genera and experimental variables (relative abundance and data transformed to base 10 logarithm), and volcano plots generated with DESeq2 to detect differentially abundant genera.

The statistical analyses for gut *Tph1*, serum 5-HT, serum tryptophan, and SCFA in feces were analyzed using unpaired t tests with Welch’s correction to determine differences between the experimental and control groups. These analyses were performed using GraphPad Prism 6.0 statistical software (La Jolla, CA, USA). The distribution of the values of the studied parameters was tested for normality using the Shapiro–Wilk test. All data are expressed as the mean ± standard error (S.E.M.) of three independent assays. Statistical significance was set at *p* < 0.05.

## 5. Conclusions

In the present study, chronic administration of EFV to healthy mice altered the gut microbiota, specifically increasing the abundance of *Bacillota* and *Dubosiella* while decreasing that of *Lactobacillus* and *Faecalbaculum*. These results may be associated with *Tph1* dysregulation in the gut and decreased serum 5-HT levels. Dysbiosis and the subsequent products of these bacteria (acetic, propionic, butyric, and valeric acids) increased. This could contribute to neuropsychiatric alterations, such as depression, caused by the chronic administration of EFV. Further studies are required to clarify whether SCFAs reach the brain and contribute to these effects. The development of innovative antidepressant treatments focused on gut microbiota transplantation could be beneficial for PLWH that consume EFV, highlighting the importance of a healthy gut microbiome for mental health and overall well-being.

## Data Availability

The original contributions presented in this study are included in the article. Further inquiries can be directed to the corresponding author.
